# Supplementary material for: Abrogation of pathogenic attributes in drug resistant Candida auris strains by farnesol
Source: PLoS One. 2020 May 11;15(5):e0233102. doi: 10.1371/journal.pone.0233102 (PMC7213696; doi:10.1371/journal.pone.0233102)
Supplement: Attachment — Submitted filename: PONE-D-19-35054_REVIEW.pdf [file pone.0233102.s001.pdf]

PLOS ONE

Abrogation of pathogenic attributes in drug resistant Candida auris strains by farnesol

--Manuscript Draft--

**Manuscript Number:**	PONE-D-19-35054
**Article Type:**	Research Article
**Full Title:**	Abrogation of pathogenic attributes in drug resistant Candida auris strains by farnesol
**Short Title:**	Farnesol inhibits pathogenicity markers in C. auris
**Corresponding Author:**	Aijaz Ahmad, Ph.D.<br>University of the Witwatersrand<br>Johannesburg, Gauteng SOUTH AFRICA
**Keywords:**	C. auris; farnesol; biofilm; efflux pump; pathogenicity markers
**Abstract:**	Candida auris , a decade old Candida species, has been identified globally as a significant nosocomial multidrug resistant (MDR) pathogen responsible for causing invasive outbreaks. Biofilms and overexpression of efflux pumps such as Major Facilitator Superfamily and ATP Binding Cassette are known to cause multidrug resistance in Candida species, including C. auris . Therefore, targeting these factors may prove an effective approach to combat MDR in C. auris . In this study, 25 clinical isolates of C. auris from different hospitals of South Africa were used. All the isolates were found capable enough to form biofilms on 96-well microtiter plate that was further confirmed by MTT reduction assay. In addition, these strains have active drug efflux mechanism which was supported by rhodamine-6-G extracellular efflux and intracellular accumulation assays. Antifungal susceptibility profile of all the isolates against commonly used drugs was determined following CLSI recommended guidelines. We further studied the role of farnesol, an endogenous quorum sensing molecule, in modulating development of biofilms and drug efflux in C. auris . The MIC for planktonic cells ranged from 62.5-125 mM and for sessile cells was 125 mM (0 h and 4 h biofilm) and 500 mM (12 h and 24 h biofilm). Farnesol inhibited biofilm formation, blocked efflux pumps and downregulated biofilm- and efflux pump-associated genes. Modulation of C. auris biofilm formation and efflux pump activity by farnesol represent a promising approach for controlling life threatening infections caused by this pathogen.
**Order of Authors:**	Vartika Srivastava
	Aijaz Ahmad, Ph.D.
**Additional Information:**	
**Question**	**Response**
**Financial Disclosure**<br><br>Enter a financial disclosure statement that describes the sources of funding for the work included in this submission. Review the submission guidelines for detailed requirements. View published research articles from *PLOS ONE* for specific examples.<br><br>This statement is required for submission and **will appear in the published article** if the submission is accepted. Please make sure it is accurate.	AA acknowledges financial support from National Research Foundation Research Development Grant for Y-Rated Researchers (RDYR180418322304; Grant No: 116339).<br><br>VS is thankful to Health Sciences Faculty Research Committee, University of the Witwatersrand for the financial support (FRC, Grant no: 001254846410151211050000000000000005254).

Powered by Editorial Manager® and ProduXion Manager® from Aries Systems Corporation

**Unfunded studies**<br>Enter: *The author(s) received no specific funding for this work.*<br><br>**Funded studies**<br>Enter a statement with the following details:<br>• Initials of the authors who received each award<br>• Grant numbers awarded to each author<br>• The full name of each funder<br>• URL of each funder website<br>• Did the sponsors or funders play any role in the study design, data collection and analysis, decision to publish, or preparation of the manuscript?<br>• **NO** - Include this sentence at the end of your statement: *The funders had no role in study design, data collection and analysis, decision to publish, or preparation of the manuscript.*<br>• **YES** - Specify the role(s) played.<br><br>* typeset	
**Competing Interests**<br><br>Use the instructions below to enter a competing interest statement for this submission. On behalf of all authors, disclose any competing interests that could be perceived to bias this work—acknowledging all financial support and any other relevant financial or non-financial competing interests.<br><br>This statement **will appear in the published article** if the submission is accepted. Please make sure it is accurate. View published research articles from *PLOS ONE* for specific examples.	The authors have declared that no competing interests exist.

Powered by Editorial Manager® and ProduXion Manager® from Aries Systems Corporation

**NO authors have competing interests**<br><br>Enter: *The authors have declared that no competing interests exist.*<br><br>**Authors with competing interests**<br><br>Enter competing interest details beginning with this statement:<br><br>*I have read the journal's policy and the authors of this manuscript have the following competing interests: [insert competing interests here]*<br><br>* typeset	
**Ethics Statement**<br><br>Enter an ethics statement for this submission. This statement is required if the study involved:<br><br>• Human participants<br>• Human specimens or tissue<br>• Vertebrate animals or cephalopods<br>• Vertebrate embryos or tissues<br>• Field research<br><br>Write "N/A" if the submission does not require an ethics statement.<br><br>General guidance is provided below. Consult the submission guidelines for detailed instructions. **Make sure that all information entered here is included in the Methods section of the manuscript.**	All the Candida auris isolates were collected with an approval by the Human Research Ethics Committee of University of the Witwatersrand (M140159) and performed according to guidelines outlined in the Helsinki Declaration.

Powered by Editorial Manager® and ProduXion Manager® from Aries Systems Corporation

**Format for specific study types**<br><br>**Human Subject Research (involving human participants and/or tissue)**<br>• Give the name of the institutional review board or ethics committee that approved the study<br>• Include the approval number and/or a statement indicating approval of this research<br>• Indicate the form of consent obtained (written/oral) or the reason that consent was not obtained (e.g. the data were analyzed anonymously)<br><br>**Animal Research (involving vertebrate animals, embryos or tissues)**<br>• Provide the name of the Institutional Animal Care and Use Committee (IACUC) or other relevant ethics board that reviewed the study protocol, and indicate whether they approved this research or granted a formal waiver of ethical approval<br>• Include an approval number if one was obtained<br>• If the study involved *non-human primates*, add *additional details* about animal welfare and steps taken to ameliorate suffering<br>• If anesthesia, euthanasia, or any kind of animal sacrifice is part of the study, include briefly which substances and/or methods were applied<br><br>**Field Research**<br><br>Include the following details if this study involves the collection of plant, animal, or other materials from a natural setting:<br>• Field permit number<br>• Name of the institution or relevant body that granted permission	
**Data Availability**<br><br>Authors are required to make all data underlying the findings described fully available, without restriction, and from the time of publication. PLOS allows rare exceptions to address legal and ethical concerns. See the PLOS Data Policy and FAQ for detailed information.	Yes - all data are fully available without restriction

A Data Availability Statement describing where the data can be found is required at submission. Your answers to this question constitute the Data Availability Statement and **will be published in the article**, if accepted.<br><br>**Important:** Stating 'data available on request from the author' is not sufficient. If your data are only available upon request, select 'No' for the first question and explain your exceptional situation in the text box.<br><br>Do the authors confirm that all data underlying the findings described in their manuscript are fully available without restriction?	
**Describe where the data may be found in full sentences. If you are copying our sample text, replace any instances of XXX with the appropriate details.**<br><br>• If the data are **held or will be held in a public repository**, include URLs, accession numbers or DOIs. If this information will only be available after acceptance, indicate this by ticking the box below. For example: *All XXX files are available from the XXX database (accession number(s) XXX, XXX.)*.<br>• If the data are all contained **within the manuscript and/or Supporting Information files**, enter the following: *All relevant data are within the manuscript and its Supporting Information files.*<br>• If neither of these applies but you are able to provide **details of access elsewhere**, with or without limitations, please do so. For example:<br><br>*Data cannot be shared publicly because of [XXX]. Data are available from the XXX Institutional Data Access / Ethics Committee (contact via XXX) for researchers who meet the criteria for access to confidential data.*<br><br>*The data underlying the results presented in the study are available from (include the name of the third party*	All relevant data are within the manuscript

and contact information or URL).<br>• This text is appropriate if the data are owned by a third party and authors do not have permission to share the data.<br><br>* typeset	
Additional data availability information:	

nuscript Click here to access/download;Manuscript;Manuscript.doc

Abrogation of pathogenic attributes in drug resistant *Candida auris* strains by farnesol

Vartika Srivastava[1], Aijaz Ahmad[1,2*]

[1]Clinical Microbiology and Infectious Diseases, School of Pathology, Faculty of Health Sciences, University of the Witwatersrand, Johannesburg, 2193, South Africa

[2]Infection Control, Charlotte Maxeke Johannesburg Academic Hospital, National Health Laboratory Service, Johannesburg, 2193, South Africa

Running Title: Farnesol inhibits pathogenicity markers in *C. auris*

Aijaz Ahmad

ORCID: https://orcid.org/0000-0003-2845-0727

*Author for correspondence

Tel: +27 11 717 2515; Fax: +2786 295 3697

Email: Aijaz.Ahmad@wits.ac.za; Aijaz.Ahmad@nhls.ac.za

Abstract

Candida auris, a decade old *Candida* species, has been identified globally as a significant nosocomial multidrug resistant (MDR) pathogen responsible for causing invasive outbreaks. Biofilms and overexpression of efflux pumps such as Major Facilitator Superfamily and ATP Binding Cassette are known to cause multidrug resistance in *Candida* species, including *C. auris*. Therefore, targeting these factors may prove an effective approach to combat MDR in *C. auris*. In this study, 25 clinical isolates of *C. auris* from different hospitals of South Africa were used. All the isolates were found capable enough to form biofilms on 96-well microtiter plate that was further confirmed by MTT reduction assay. In addition, these strains have active drug efflux mechanism which was supported by rhodamine-6-G extracellular efflux and intracellular accumulation assays. Antifungal susceptibility profile of all the isolates against commonly used drugs was determined following CLSI recommended guidelines. We further studied the role of farnesol, an endogenous quorum sensing molecule, in modulating development of biofilms and drug efflux in *C. auris*. The MIC for planktonic cells ranged from 62.5-125 mM and for sessile cells was 125 mM (0 h and 4 h biofilm) and 500 mM (12 h and 24 h biofilm). Farnesol inhibited biofilm formation, blocked efflux pumps and downregulated biofilm- and efflux pump-associated genes. Modulation of *C. auris* biofilm formation and efflux pump activity by farnesol represent a promising approach for controlling life threatening infections caused by this pathogen.

Keywords: *C. auris;* farnesol; biofilm; efflux pump; pathogenicity markers

Introduction

Candida auris has now well evolved MDR pathogen, which has caused serious outbreaks in several continents. It was first isolated from external ear of a Japanese patient in 2009 [1] and within a decade infection caused by *C. auris* has spread rapidly across six continents [1, 2]. Centers for Disease Control and Prevention (CDC) has declared *C. auris* as a global threat with a report of causing several outbreaks in different countries, including United States (https://www.cdc.gov/fungal/candida-auris/tracking-c-auris.html). *C. auris* is causing serious bloodstream infections and other infections ranging from meningitis, bone infections, surgical wound infections and urinary tract infections have been reported in hospitals [3]. *C. auris* infections are stubborn because it is resilient to the available antifungal drugs including fluconazole (first-line antifungal drug) and amphotericin B. In one of the reports, CDC has analyzed antifungal susceptibility profile of different *C. auris* isolates and it was reported that almost all the isolates were resistant to azoles (fluconazole) and 1/3 of isolates remain unaffected to polyenes (amphotericin B). In 2016, Infectious Diseases Society of America (IDSA) Clinical Practice Guidelines for Candidiasis has recommended Antifungal susceptibility testing (AFST) for all clinically-relevant *Candida* isolates. Furthermore, it was suggested that any *Candida* isolate with antifungal resistance ≥ 1 and with uncertain identity should be further tested for confirmation of *C. auris* [4]. In a study, out of 54 *C. auris* isolates from five countries, FLZ resistance was reported in 93 % isolates, AmB resistance in 35 % isolates, and echinocandins resistance in 7% *C. auris* isolates (around 41 % *C. auris* isolates were found resistant to ≥ 2 antimycotic class of drugs) [5]. Similarly, a recent report clears scenario of *C. auris* resistance in the U.S., 86 %, 43 % and 3 % of first 35 patients were resistant to fluconazole, amphotericin B, and echinocandins respectively [6].

Whereas, echinocandins class of drugs was found active against most of the isolates of *C. auris*; however, echinocandin resistance in patients have been reported recently [7]. Researchers have reported multidrug resistance among *C. auris* isolates as a common phenomenon, severely restraining its treatment possibilities [5]. In South Africa, the first instance of infection caused by *C. auris* was reported in the 2014 and around 1,700 cases were detected between 2012 and 2016. Currently, *C. auris* is a widespread problem as it is found in almost 100 hospitals across South Africa with a vast majority of cases been reported in Gauteng [8].

Increasing prevalence of *C. auris* infection worldwide especially in South Africa motivated us to study pathogenic traits of this species. Farnesol, a first quorum sensing (QS) molecule identified in eukaryotic microorganisms [9], play an important role in an array of biological functions such as virulence, biofilm formation, and competence [10]. Numerous studies have reported the effect of farnesol on *C. albicans* growth and pathogenesis [11-13]. In *C. albicans*, farnesol inhibits the dimorphism [9] that prevent its establishment in different environmental conditions [14], it has antioxidant effects [15] and it inhibits transporters [16]. Additionally, farnesol showed low cytotoxicity without genotoxic effects [17]. With this background, we emphasized to study the effect of farnesol on growth, biofilms and reversal of drug resistance in different *C. auris* isolates.

Methods

***Candida* isolates**

In this study, ~~26 isolates of *Candida* spp. including~~ 25 *C. auris* and 1 *C. albicans* (SC5314) were used (Table 1). All the 25 clinical *C. auris* strains were obtained from the Division of Mycology, National Institute of Communicable Diseases, Johannesburg, South Africa. All these isolates

were collected with an approval by the Human Research Ethics Committee of University of the Witwatersrand (M140159) and performed according to guidelines outlined in the Helsinki Declaration. Identification was performed using Matrix-assisted laser desorption/ionization time-of-flight (MALDI-TOF) technique. The isolates were stored in glycerol stock at -80 °C until required.

which machine? which database?

Table 1: List of *Candida* isolates

***Candida* spp. isolates used in study**	
C. auris MRL 6326	*C. auris* MRL 3499
C. auris MRL 6183	*C. auris* MRL 6194
C. auris MRL 4888	*C. auris* MRL 6005
C. auris MRL 6015	*C. auris* MRL 6057
C. auris MRL 6333	*C. auris* MRL 5762
C. auris MRL 4587	*C. auris* MRL 6173
C. auris MRL 6334	*C. auris* MRL 5765
C. auris MRL 3785	*C. auris* MRL 2397
C. auris MRL 6059	*C. auris* MRL 5418
C. auris MRL 4000	*C. auris* MRL 6277
C. auris MRL 6065	*C. auris* MRL 6339
C. auris MRL 2921	*C. albicans* SC5314
C. auris MRL 6125	
C. auris MRL 6338	

place in numerical order in text.

already mentioned

Antifungal susceptibility profiles

The susceptibility profile of *C. auris* isolates were established by broth microdilution assay as per the recommended guidelines of Clinical and Laboratory Standards Institute (CLSI) reference document M27-A3 [18]. Briefly, stock solution of AmB was prepared by using 1% dimethyl sulfoxide (DMSO) and the range of concentration tested was 16 – 0.031 µg/ml. Similarly, FLZ stock solution was prepared using deionized water and the range of concentration tested ranged from 1000 – 1.0 µg/ml. Farnesol stock solution was prepared with 1% DMSO and the concentration tested ranged from 250 – 0.48 mM. The drugs and farnesol at desired concentrations (100 µl) were introduced in designated wells of 96-well microtiter plate containing 100 µl of *C. auris* cell suspension (5.0×10^6 CFU/ml). These plates were incubated at 37 °C for 48 h without shaking. In every set of experiment, cell free (sterility) and drug free (growth) controls were included for each *C. auris* isolates and all the isolates were tested in triplicate. *C. albicans* SC5314 was kept as a standard laboratory control in each test performed. Observation was made visually as well as by employing 3-(4,5-Dimethyl-2-thiazolyl)-2,5-diphenyl-2H-tetrazolium bromide (MTT) reduction assay [19]. Briefly, a stock solution of MTT in Phosphate Buffer Saline (PBS) (5 mg/ml, filter sterilized and diluted 1:5 with pre-warmed sterile PBS). After 48 h incubation, 50 µl of MTT solution was added to each well of the microtiter plate and was incubated for 5 h at 37 °C. Subsequently, 100 µl of DMSO was added to solubilize the MTT-formazan product, which was measured at 490 nm by using a microplate reader (iMark, BioRad). The MICs were defined as the lowest concentration of AmB farnesol that resulted in the complete inhibition of growth whereas 80 % inhibition was considered in case of FLZ.

***Candida auris* biofilm formation**

Biofilm formation by *Candida* spp. on medical devices is very common problem and life threatening for patients. Different clades of *C. auris* have also been reported to produce biofilm and therefore we studied the biofilm forming capability of South African clade *C. auris* isolates. The method adapted to evaluate biofilm formation by *C. auris* isolates has been described previously [19]. The metabolic activity of *C. auris* biofilm was also compared with *C. albicans* SC5314 biofilms. The metabolic activity was also compared among *C. auris* isolates and those with higher readings were selected for further investigation (drug efflux and accumulation studies as well as for molecular analysis).

Effect of farnesol on development of *C. auris* biofilms

To evaluate the activity of farnesol on sessile cells of *C. auris* and later development of biofilms, a method described previously was followed [19]. Briefly, 100 µl standardized cell suspensions (5.0×10^6 CFU/ml) were inoculated into predetermined wells of 96-well microtiter plates followed by incubation at 37 °C for 0, and 4 h. After incubation the growth medium was removed followed by thorough washing with sterile PBS. After removal of non-adherent cells, different concentrations (500 mM to 0.488 mM) of farnesol were added to the wells of microtiter plate. To check the effect of farnesol on adherence and biofilm formation, farnesol and standardized suspension were added together to microtiter plate (zero time/preincubation) and incubated for 48 h. Furthermore, to see the effect on 4 h mature biofilms, cells were incubated under biofilm forming conditions for 4 h and then sessile cells were removed, washed gently with sterile PBS and farnesol was added to predetermine wells and incubated for 48 h. Metabolic activities of the biofilms were measured using MTT reduction assay. Briefly, a stock solution of MTT (as described in section 2.2) was prepared. After biofilm formation, 50 µl of MTT solution was added to each well of the 96-well microtiter plate and was incubated for 5 h at 37 °C.

Subsequently, MTT was removed and 100 µl of DMSO was added to solubilize the MTT-formazan product, which was measured at 490 nm by using a microplate reader (iMark, BioRad).

Effect of farnesol on mature biofilms

C. auris biofilms were allowed to grow for 12 and 24 h at 37 °C under favorable biofilm forming conditions. The growth medium was removed, and biofilm was washed gently with sterile PBS. Farnesol (500 mM to 0.488 mM) was added to the predefined wells of microtiter plates and further incubated for 24 h at 37 °C. The metabolic activity of treated and untreated biofilms was assessed by MTT reduction assay (as described in section 2.4). The lowest concentration of farnesol where we reported ≥ 90 % destruction in mature biofilm was recorded. Furthermore, biofilm inhibitory concentrations (BIC) were defined as the lowest concentration of farnesol where we report inhibition (≥90%) compared to the growth control.

Confocal laser scanning microscopy (CLSM)

To further confirm the effect of farnesol on *C. auris* biofilm, CLSM was done. *C. auris* strain MRL 5765 was allowed to grow on glass coverslips in 6-well microtiter plates under biofilm forming conditions. Farnesol (BIC) was administered in designated wells at different time points (4 h, 12 h and 24 h) except the growth control wells (untreated cells). The plates were further incubated for 24 h at 37 °C. Following incubation, the planktonic cells were aspirated and biofilms were gently washed twice with PBS and stained with fluorescent dye FUN-1 (Invitrogen, Thermo Fisher Scientific, ZA) and concanavalin A (ConA)-Alexa Fluor 488 conjugate (Invitrogen, Thermo Fisher Scientific, ZA). For staining, the coverslips were transferred to a new 6-well microtiter plate and incubated with 2 ml PBS containing FUN-1 (10 µM) and ConA-Alexa Fluor 488 conjugate (25 µg/ml) for 45 min at 37 °C in dark. FUN-1

(excitation wavelength = 543 nm and emission wavelength = 560 nm) is a vital dye and only live cells are capable of transporting it to the vacuole and result into orange-red cylindrical intra-vacuolar structures (CIVS) whereas in dead cells FUN-1 remain in the cytosol and fluoresces yellow-green [20]. ConA (excitation wavelength = 488 nm and emission wavelength = 505 nm) on the other hand fluoresces bright green when binds to α-mannopyranosyl and α-glucopyranosyl residues present in cell wall and biofilm matrix. After incubation with fluorescent dyes the glass coverslips were flipped on glass plates and stained biofilms were observed using a Zeiss Laser Scanning Confocal Microscope (LSM) 780 and Airyscan (Carl Zeiss, Inc.). Multitrack mode was used to collect the images of green (ConA) and red (FUN-1) fluorescence simultaneously. The thickness or volume of whole biofilm was determined by collecting Z-stack picture and the distances between first and last fluorescent confocal plane was defined as biofilm thickness [21].

Extracellular Rhodamine 6G efflux assay

Extracellular efflux of Rhodamine 6G (R6G) from *C. auris* cells were evaluated as described previously [22] with some minor adjustments. For this study four *C. auris* isolates (MRL 4000, MRL 5765, MRL 5762, and MRL 6057) were selected and *C. albicans* SC5314 was used as standard for efflux activity. Briefly, *Candida* cells were grown on Sabouraud Dextrose Agar (SDA) plates for 24 h at 37 °C. The cells (5.0×10^6 CFU/ml) were inoculated in 50 ml growth media (SDB) for 8 h at 37 °C. Post incubation media was centrifuged (3000 rpm), washed with 25 ml PBS (without glucose) at least two times. The washed cells were resuspended in sterile glucose-free PBS (2% cell suspension). The cells were further incubated in 50 ml PBS containing 2-deoxy-D-glucose (5.0 mM) and 2,4 dinitrophenol (5.0 mM) for 45 min, resulting in de-energizing of cells. Followed by de-energization the cells were washed and again resuspended in glucose-free PBS (2% cell suspension), R6G (final concentration of 10 μM) was added to this

resuspension and incubated for 40 min at 37 °C. After incubation cells were again washed and resuspended in glucose-free PBS (2% cell suspension). Samples (2 ml) were withdrawn at definite intervals (0, 5, 10, 15, 20 min). After harvesting samples were pelleted at 3,000 rpm and optical density of supernatant was recorded at 527 nm. To study the energy dependent R6G efflux, glucose (0.1 M) was added after 20 min incubation to the cells resuspended in glucose-free PBS. The absorbance was recorded till 60 min of incubation with glucose and the last reading was recorded after overnight (20 h; 1200 min) incubation. Positive as well as negative controls were included in all the experiments. The standard concentration curve of R6G was prepared for determining the actual concentration of R6G effluxed.

For competition assays, yeast cells were exposed for 2 h to different concentration of farnesol (0.5 × MIC and MIC). Post exposure the cells were pelleted (3000 rpm) and washed twice with sterile PBS (without glucose). Thereafter treated cells were de-energized and then equilibrated in R6G as stated above. Samples (2 ml) were withdrawn at predetermined time points (0, 5, 10, 15, 20 min), centrifuged (3,000 rpm) and absorbance of supernatant was recorded at 527 nm. The estimation of energy dependent R6G efflux was done by adding glucose (0.1 M) after 20 min incubation to the resuspended cells and reading were recorded till 60 min and last reading was recorded after 1200 min of incubation. Positive as well as negative controls were included in all the experiments. The standard concentration curve of R6G was prepared for determining the actual concentration of R6G effluxed.

Intracellular Rhodamine 6G accumulation assay

Intracellular accumulation assay was executed as discussed earlier with minor modifications [22]. Briefly, *C. auris* isolates (MRL 6057, MRL 4000, MRL 5762, and MRL 5765) cells were

grown overnight in SDB medium at 37 °C. After incubation cells were centrifuged and washed twice in sterile PBS and re-inoculated in sterile SDB broth supplemented with farnesol (at 0.5 × MIC and MIC) for 2 h at 37 °C. Post incubation, cells were pelleted (3000 rpm) and given sterile PBS wash. The washed cells (5.0 × 10^6 CFU/ml) were resuspended in sterile PBS (1.0 ml) supplemented with glucose (2%) and R6G (4 µM) and then incubated for 30 min at 37 °C. Post incubation cells were washed twice with cold sterile PBS and the pellet was used for fluorescence microscopy.

Real Time PCR

The mRNA expression level for *CDR1*, *CDR2*, *SNQ2*, *HYR3*, *IFF4*, *PGA7*, *PGA26*, *PGA52*, *MDR1*, *MDR2* and *ACT1* (housekeeping gene) were measured using RT-qPCR. The method was taken from previous study described elsewhere [23]. Briefly, *C. auris* isolates (MRL 6057, MRL 4000, MRL 5762 and MRL 5765) were incubated overnight at 37 °C SDB medium. Overnight cultures were centrifuged (3000 rpm) resulting a pellet, which was resuspended in sterile SDB broth (10 ml) containing farnesol (MIC) and then incubated at 37 °C for 2 h. After exposure time centrifugation (3000 rpm) was done, supernatant was discarded and pellet was used for RNA extraction. RNA was extracted by using RNA MiniPrep kit (Inqaba Biotechnical Industries (Pty) Ltd) as instructed by the manufacturer. The Nano Drop 2000 spectrophotometer (Thermo Scientific) was used to determine concentrations of isolated RNAs. Purity of RNAs was assessed by determining A_{260}/A_{280} ratio and a ratio above 2 was used for further experiment (qRT-PCR). cDNA was synthesized using Lasec SA (Pty) Ltd cDNA kit following manufacturer's instructions. PCR master mix and PowerUp SYBR Green Master Mix (Applied Biosystems) were used to amplify *C. auris* genes from cDNA by Light Cycler Nano Real-Time PCR system (Roche). **Table 2** enlists the primers (both forward and reverse) used for amplification and

experimental conditions were as follows: UDG activation at 50 °C for 2 min (Hold), Dual-lock DNA polymerase at 95 °C for 2 min (Hold), 40 cycles of denaturation at 95°C for 15 sec, annealing at 53 °C for 15 sec, and extension 72 °C for 1 min. Dissociation curve conditions (melt curve stage) were as follows: Pre-melting at ramp rate of 1.6 °C/sec, 95 °C and 15 sec; Melting at ramp rate of 1.6 °C/sec, 60 °C and 1 min; Melting at ramp rate of 0.15 °C/sec, 95 °C and 15 sec. The dissociation curve and CT values were determined using the Light Cycler Nano system. The gene expression was quantified and analyzed with respect to the housekeeping gene *ACT1* using formula $2^{-\Delta\Delta CT}$. The relative change in expression was estimated by normalizing to housekeeping gene (*ACT1*).

Table 2: Nucleotide sequences for primers (5′—3′)

Gene	Forward primer	Reverse primer
**CDR1*	GAAATCTTGCACTTCCAGCCC	CATCAAGCAAGTAGCCACCG
**MDR1*	GAAGTATGATGGCGGGTG	CCCAAGAGAGACGAGCCC
SNQ2	ATCACCGAGGAATTGAGCAC	TCAACCTGTGAGCTTGATGC
HYR3	CTGGTTTGACCTTCGTGGAT	GGCAGAGGTGACGTAGAAGC
IFF4	AATGGTGCTGGTTGTGTGAA	AGTGAACCCAAGGTTGATGC
PGA26	CCACGAACCTCCAAACAAGT	TGGTCACTGTGAGGGTGGTA
PGA7	GGCAGACTTTTCAGCTTTGG	AATCAATTTCCCGTTTGCAG
PGA52	ACGAACACACCGTTGAATGA	AGTGCCATCTTGAGCGCTAT
**ACT1*	GAAGGAGATCACTGCTTTAGCC	GAGCCACCAATCCACACAG
**CDR2*	GTCAACGGTAGCTGTGTG	GTCCCTCCACCGAGTATGG
**MDR2*	GGCGAGCTGTTGAGAATGTG	CTTCATGGCTTGCAACCTTC

ALS5	GCGATCCAATTTTGGAAGAA	GGTGCATCCCTATCTGAGGA

*Primers for the gene, *CDR1, CDR2, MDR1, MDR2* and *ACT1* were obtained from Rybak *et al*., 2019 [23]. Whereas, other primer sequences were designed by online Primer3web version 4.1.0.

Statistics

All the data and graphs were made and statistically analyzed using GraphPad prism version 5.01. All the experiments were carried out in triplicates, and the data obtained were presented as means ± standard error of the mean. Two-way ANOVA was used to compare untreated control with treated groups and P value less than 0.05 was considered significant.

Results and Discussion

Antifungal susceptibility testing

All the clinical isolates of *C. auris* used in the present study were found sensitive to the farnesol within the MIC range of 62.5 - 125 mM. MIC values for AmB ranged from 0.125 - 4.0 µg/ml whereas for FLZ the MIC values ranged from 16 – 500 µg/ml **(Table 3)**. As there are no confined cutoff values to differentiate susceptible and resistant *C. auris* isolates against these drugs, it would have been inappropriate to categorize these isolates. However, CDC has established arbitrary breakpoints for *C. auris*, which were set at ≥ 32 µg/ml and ≥ 2 µg/ml for FLZ and AmB, respectively [8, 24]. Based on these cutoff values, all the tested *C. auris* isolates except three (MRL 3785, MRL 3499, MRL 2397) were FLZ resistant whereas five *C. auris* isolates (MRL 2921, MRL 4000, MRL 5765, MRL 5762 and MRL 6057) were found resistant to AmB. Recent studies have also confirmed that *C. auris* isolates are usually resistant or less susceptible to azoles [25-27]. Furthermore, lower susceptibility of *C. auris* isolates against AmB is also in agreement with previous studies, where high AmB MICs for *C. auris* isolates was

reported [28-30]. Inhibitory and modulatory effects of farnesol in *C. albicans* and other non-albicans has already being studied and its impact on biofilm formation, efflux pumps, and other virulence attributes is well established [11, 31-33]. However, this study for the first time reported inhibitory effect of farnesol on *C. auris* isolates.

Table 3: MIC values for AmB, FLZ and farnesol against isolates of *C. auris*.

***C. auris* isolates**	**AmB (µg/ml)**	**Fluconazole (µg/ml)**	**Farnesol (mM)**	***C. auris* isolates**	**AmB (µg/ml)**	**Fluconazole (µg/ml)**	**Farnesol (mM)**
MRL 6326	0.25	125	125	MRL 3499	0.5	16	62
MRL 6183	0.25	250	125	MRL 6194	0.25	125	125
MRL 4888	1.0	500	125	MRL 6005	1.0	500	125
MRL 6015	0.25	62	125	MRL 6057	4.0	125	125
MRL 6333	0.5	125	125	MRL 5762	2.0	500	125
MRL 4587	0.5	32	125	MRL 6173	0.25	32	125
MRL 6334	0.5	250	125	MRL 5765	2.0	500	125
MRL 3785	0.125	16	62	MRL 2397	1.0	16	125
MRL 6059	0.5	125	125	MRL 5418	0.5	500	125
MRL 4000	2.0	250	125	MRL 6277	0.5	125	125
MRL 6065	1.0	125	125	MRL 6339	0.5	250	125
MRL 2921	2.0	250	125				
MRL 6125	0.25	62	125				
MRL 6338	0.25	125	125				
